# Expanding the landscape of nucleotide excision repair disorders: from discovery to therapy

**DOI:** 10.1172/JCI199822

**Published:** 2025-11-17

**Authors:** Arjan F. Theil, Jan H.J. Hoeijmakers

**Affiliations:** 1 Department of Molecular Genetics, Erasmus MC Cancer Institute, Erasmus University Medical Center, Rotterdam, Netherlands.; 2 University Hospital of Cologne, CECAD Forschungszentrum, Institute for Genome Stability in Aging and Disease, Köln, Germany.; 3 Princess Maxima Center for Pediatric Oncology, Oncode Institute, Utrecht, Netherlands.

## Abstract

DNA damage and repair are central to the onset of cancer, aging, and aging-related diseases. Rare genetic defects in the nucleotide excision repair pathway, such as those causing the cancer-prone disorder xeroderma pigmentosum (XP) or the progeroid condition Cockayne syndrome, highlight the dramatic consequences of unrepaired DNA lesions. In this issue of the *JCI*, two related papers from Ogi and coworkers — Fassihi et al. and Nakazawa et al. — describe a new XP clinical entity, XP-J, linked to a pathogenic variant in the p52 subunit of the transcription-repair complex TFIIH. The studies’ characterization of XP-J and the p52ΔC variant opened unexpected possibilities to ameliorate the molecular defect in another subunit of TFIIH that causes a different, more severe repair syndrome: trichothiodystrophy. This commentary provides a broader historical, medical, and molecular context for the intricate genotype-phenotype relationship between compromised repair and its clinical consequences and discusses next steps for the advances reported.

## Introduction

Nucleotide excision repair (NER) is a versatile DNA repair pathway that excises base pair-disrupting and transcription-stalling lesions, such as UV-photoproducts and numerous chemical adducts (1). The NER pathway comprises two subpathways: Global Genome NER (GG-NER) (2), which removes distorting damage anywhere in the genome, and Transcription-Coupled NER (TC-NER) (3), which eliminates DNA lesions when they are physically stalling transcription. Rare genetic defects in NER give rise to remarkably different disorders, all sharing sun (UV) hypersensitivity. Xeroderma pigmentosum (XP) results from deficiencies primarily affecting GG-NER and is characterized by extreme cancer predisposition (4), with patients exhibiting striking photosensitivity, freckling and early pigmentary changes, ocular abnormalities (e.g., photophobia), and a greater than 1000-fold risk of developing skin cancers (e.g., basal and squamous cell carcinoma, melanoma). In contrast to XP, Cockayne syndrome (CS) arises from compromised TC-NER and is marked by photosensitivity and many severe progressive manifestations of premature aging, including neurodegeneration, early cessation of development and growth, brain abnormalities (e.g., calcification, demyelination), ophthalmological problems (e.g., cataracts), hearing loss, and microcephaly, but does not exhibit cancer predisposition (5). Trichothiodystrophy (TTD) is a more heterogeneous disorder caused by defects involving the basal process of gene expression and, when caused by defects in the dual functional transcription-repair complex TFIIH, both GG- and TC-NER are affected as well (6), adding XP and CS symptoms to the TTD spectrum. TTD-specific features include developmental delay, growth failure, and microcephaly, in addition to the typifying hallmarks of brittle hair and nails, and cutaneous manifestations (e.g., ichthyosis, scaly skin).

The multifunctional 10-subunit TFIIH complex locally unwinds the DNA for RNA-polymerase II binding in transcription initiation and for lesion verification and excision in both NER subpathways (7). Because of the multiple engagements of TFIIH, different mutations in a single TFIIH subunit can trigger any of the clinical entities described above (TTD, XP and CS), depending on the mutation’s effect on, respectively, gene transcription, GG-NER, or TC-NER (or its combined effects). Hence, TFIIH defects are associated with an exceptionally wide and heterogenous spectrum of clinical symptoms. This makes strict classification into XP, CS, and TTD often difficult, supporting NER disorders as a continuum (7, 8).

In this issue of the *JCI*, Fassihi et al. (9) report discovering a ninth XP complementation group, XP-J, caused by mutations in the p52 subunit of TFIIH, encoded by *GTF2H4*. The C-terminal truncating variant is designated in the study as p52ΔC. Remarkably, XP-J’s discovery comes 50 years after the prior XP gene discovery. The group extended these findings in a second paper in this issue, in which Nakazawa et al. (10) uncovered that p52ΔC opens unexpected opportunities for therapy of a defect in another TFIIH subunit, p8 (encoded by *GTF2H5*), which causes the clinical entity TTD-A. TTD-A is associated with a more severe phenotype than XP-J because the pathogenic p8 variant destabilizes the entire TFIIH complex. Paradoxically, introducing the p52ΔC mutation in p8-defective cells stabilized TFIIH again, which — if successfully translated at the patient level — may convert the severe phenotype associated with TTD-A defect to the milder XP-J phenotype, although there are still unknowns.

## A historical perspective

Cleaver’s discovery of XP as a human DNA repair disorder in 1968 kickstarted an extraordinary journey in human genetics (11). Soon thereafter, cell fusion by Bootsma and colleagues revealed that XP is not a monogenetic entity (12) and, together with others, identified in total seven complementation groups XP-A to XP-G, each caused by mutations in different repair genes. The last exceptional group, XP-V, did not carry a defect in NER, but rather carried a defect in a then ill-defined process called postreplication repair (13). However, it was apparent that XP also displayed considerable clinical heterogeneity, and when NER defects were also found to underlie two other very different diseases, CS and TTD, the spectrum of NER disorders further expanded.

Over the next 25 years, molecular cloning and functional assays identified all key NER genes, including those in which deficiency causes XP-A through -G, culminating in the outline of the GG- and TC-NER reaction mechanisms just before closure of the twentieth century (14). However, now, half a century after characterization of XP-V, the Ogi research group has discovered a patient harboring a mutation that represents a new XP group, XP-J, using whole genome sequencing supported by functional assays. The mild XP-J phenotype characterized in this patient is caused by biallelic mutations in the *GTF2H4* gene (encoding p52), a TFIIH subunit, thereby expanding the list of TFIIH-associated disease genes, which already included *ERCC2* (XP-D), *ERCC3* (XP-B), and *GTF2H5* (TTD-A). It is tempting to speculate that identification of disease-linked variants in more TFIIH-core subunit genes will follow, *GTF2H1* (encoding p62) and *GTF2H3* (encoding p34) being strong candidates. Mutations in the remaining TFIIH core subunit *GTF2H2* (encoding p44) are unlikely to be associated with disease because its gene is duplicated.

## Classification of NER-related disorders

**Figure 1:**
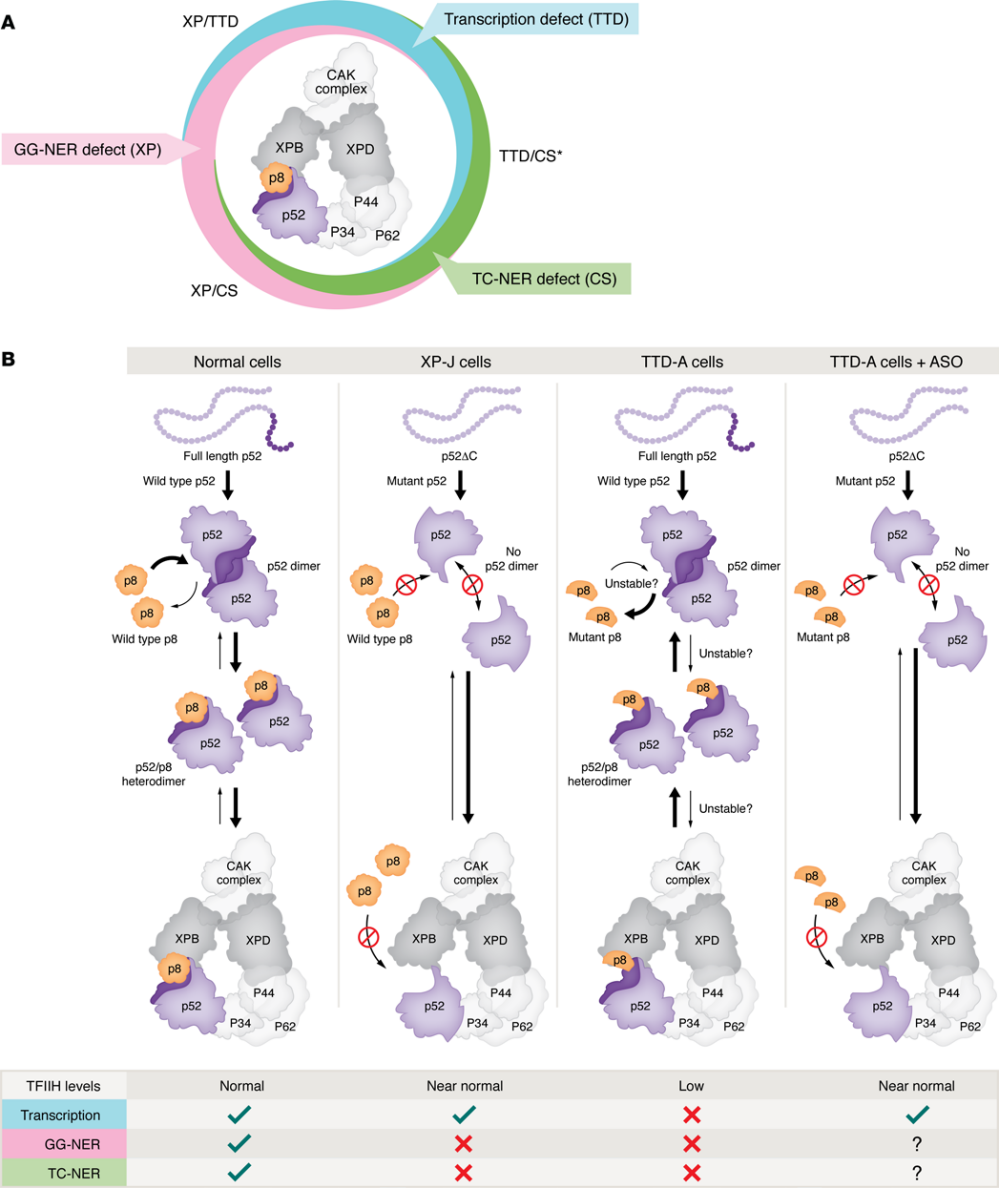
NER-associated disorders, TFIIH function, and a potential intervention for TTD-A. (**A**) Classification of XP, CS, and TTD in relation to functional defects in TFIIH. The primary clinical classifications overlap (XP/TTD, TTD/CS, XP/CS) based on which functions are affected. (* denotes that combined TTD/CS phenotype may best capture the clinical spectrum, rather than solely TTD). In TTD, TFIIH stability is affected, causing exhaustion of TFIIH, and, hence, of gene expression prior to completion of terminal differentiation. NER-disease–associated TFIIH subunits are indicated in dark gray (XPB, XPD) and orange (p8, defective in TTD-A cells), while purple highlights p52, which the related studies have associated with the NER-disease XP-J (9,10). (**B**) The stability and functions of the TFIIH complex critically depend on the p52–p8 interaction, where p8 is thought to remodel p52 homodimer to enable proper TFIIH assembly. In normal cells (leftmost column), WT p8 forms a stable heterodimer with the C-terminal domain of p52, enabling assembly with other TFIIH subunits, generating a fully functional TFIIH complex to carry out transcription, GG-NER, and TC-NER. In XP-J cells (second column), a mutation in GTF2H4 produces a C-terminally truncated p52 protein (p52ΔC), lacking the domain for p52 homo- or p52–p8 heterodimerization. p52ΔC remains capable of interacting with TFIIH, leading to near-normal levels of a p8-deficient TFIIH complex with impaired GG- and TC-NER but unaffected transcriptional functions (9). In TTD-A cells (third column), a mutation leads to a partially functional p8 protein, resulting in unstable p52–p8 heterodimers that impair integration into TFIIH. The resulting p8- and p52-deficient TFIIH complex becomes unstable, leading to low TFIIH levels and impaired transcription, GG-NER, and TC-NER functions. Nakazawa et al. adopted a strategy to intervene in TTD-A cells using ASOs to modulate p52 splicing (rightmost column): treatment of TTD-A cells with ASOs induced a XP-J–like p52ΔC. p52ΔC lacks the domain for interaction with the TTD-A p8 mutant protein, but can directly interact with TFIIH, restoring near-normal TFIIH levels and normal transcription. Thus, expression of p52ΔC can convert the severe TTD-A phenotype into the milder XP-J phenotype (10). The effect on GG- and TC-NER functions requires follow-up investigation.

The clinical complexity arising from the phenotypic differences between XP, CS, TTD, and their combined disorders can be explained on the basis of which process(es) is/are affected and to what extent: GG-NER in XP, TC-NER in CS, and transcription initiation for TFIIH mutations. For instance, in patients with XP-C and XP-E, the specific GG-NER defect leaves many distorting lesions in the genome unrepaired. However, since TC-NER is unaffected, cells can still transcribe their genes and survive with higher levels of damage in the nontranscribed DNA. This substantially increases accumulation of mutations and explains the extreme risk of skin cancer in XP. Conversely, in CS-A and CS-B patients, the selective defect in TC-NER hampers gene transcription at low levels of lesions, triggering functional decline and eventually cell death and tissue degeneration, explaining the premature aging in CS. The role of TFIIH in both GG-NER and TC-NER explains the XP and CS symptoms associated with, for instance, patients with XP-D, depending on the degree to which GG-NER and TC-NER are affected. The additional non-NER role of TFIIH in transcription initiation adds the TTD-specific features of brittle hair, brittle nails, and cutaneous abnormalities (e.g., ichthyosis) to the clinical spectrum for patients with TFIIH mutations. These features are caused by the instability of the mutant TFIIH complex, leading to premature abortion of terminal differentiation in hair, nails, and skin (Figure 1A).

Within the TFIIH clinical continuum, the patient harboring the p52ΔC variant presented at age 6 with typical XP features such as photosensitivity and hyperpigmentation, indicating a GG-NER defect, as confirmed by refs. 9 and 10. In view of the extreme skin cancer susceptibility of XP, it will be important to closely screen the patient for early-stage cutaneous abnormalities. The confirmed defect in TC-NER, indicative of CS, is clinically significant given the features of microcephaly and mild developmental delay, like in the case of the other TFIIH-associated CS and TTD disorders. Due to the progressive nature of these disorders and the patient’s young age, it is difficult to predict whether and to what degree CS/TTD features will manifest in the future.

## Nutritional and lifestyle interventions shield against DNA damage

As DNA damage constantly accumulates, DNA repair–deficient patients face progressive symptoms and therefore need close monitoring for optimal care. Unfortunately, no cure exists. While therapeutic interventions are eagerly awaited, lifestyle and nutritional strategies may also serve as an effective first line of defense. One obvious strategy would be reducing DNA damage as the root cause. In case of XP, for which skin manifestations are the most severe, this can be achieved by strict sun protection, strongly reducing skin cancers (15).

As for the premature aging features of CS, mouse models have provided evidence for unexpected but very promising interventions involving food. A reduction in calorie intake tripled lifespan and markedly delayed all features of accelerated aging in TC-NER–deficient progeroid mice, most notably their lethal neurodegeneration (16). This is consistent with the notion that dietary restriction, by triggering a very potent protective ‘survival’ response, is the only robust, universal antiaging intervention promoting longevity in species ranging from yeast and worms to mammals, including nonhuman primates (17). This survival response suppresses growth but boosts defense and resilience mechanisms (e.g., upregulating antioxidants), and redesigns metabolism, reducing endogenous DNA damage. Thereby, caloric restriction as a nutritional intervention diminishes transcription stress caused by unrepaired DNA lesions, physically stalling transcription, explaining its strong antiaging effect in the CS mouse mutants (16, 18). These findings stress the importance of controlled nutrition for CS (19).

Besides quantity, obviously the substances consumed are important for NER and other DNA repair disorders, particularly regarding genotoxins: e.g., acetaldehydes from alcohol, aflatoxins from contaminated grains, or polycyclic aromatic hydrocarbons from smoke, which may escape GG-NER but stall transcription (20). Thus, diet and lifestyle directly shape disease outcome and are especially important in the setting of DNA repair deficiency.

## Future therapeutic perspectives and clinical consequences

The ultimate cure for genetic disorders is correcting the underlying mutation at the DNA level. While CRISPR/Cas–based strategies advance remarkably fast and already show promise in several diseases, their application to systemic disorders such as XP, CS, and TTD is still remote. Major obstacles include safe and efficient delivery to all tissues (e.g., across the blood-brain barrier) and prevention of unintended off-target effects (e.g., carcinogenesis). Meanwhile, approaches acting at the promoter, RNA, or protein level — such as small-activating RNA (saRNA), small interfering RNA (siRNA), or allosteric modulators — may offer more feasible alternatives.

In this context, antisense oligonucleotides (ASOs) provide a highly versatile platform for tailored therapies, which allows precise modulation of gene expression parameters. The related study by Nakazawa et al. (10) demonstrated ingeniously that therapeutically induced, alternative splicing of *GTF2H4* in the described XP-J patient can be used for transcomplementation of a mutation in another TFIIH subunit, p8, that causes more a severe TTD-A phenotype (21). Inspired by the finding that the C-terminally truncated p52 protein in XP-J stabilizes the TFIIH complex even in the absence of p8 subunit binding (Figure 1B, left columns), they designed ASOs to replicate this effect in p8-deficient cells by promoting intron retention in the p52ΔC pre-mRNA. The retained intron produced a truncated p52ΔC protein similar to that in XP-J cells, thereby stabilizing the TFIIH complex and partially bypassing the TFIIH instability caused by the p8 defect (Figure 1B, right columns).

TFIIH instability appears to be a common driver of TTD (6, 22). Increasing TFIIH levels and transcriptional capacity in p8-deficient cells therefore represents a promising strategy. However, there are still unknowns. It will be important to find out whether expressing the C-terminally truncated p52 variant in the setting of pathogenic p8 mutations also restores TFIIH functions in the GG- and TC-NER subpathways. Restoring TFIIH levels while simultaneously preventing the binding of still partially functional p8 may introduce complications (23), as p8 may have additional functions. Therefore, the next step is to evaluate ASO-mediated interventions in model organisms, such as mice, as an essential stepping stone toward therapy. Together, these advances will clear a path for precision therapies that may transform the prognosis for patients across the entire spectrum of NER disorders.

## Funding support

This work is the result of NIH funding, in whole or in part, and is subject to the NIH Public Access Policy. Through acceptance of this federal funding, the NIH has been given a right to make the work publicly available in PubMed Central.

The funders played no role in study design, data collection, analysis and interpretation of data, or the writing of this manuscript.

- National Institute of Health (NIH)/National Institute of Aging (NIA) (P01 AG017242; DNA repair, mutations and cell aging).
- European Research Council Advanced Grant Dam2Age.
- Dutch research organization ZonMW Memorabel project ID733050810, ONCODE (Dutch Cancer Society).
- Deutsche Forschungsgemeinschaft (DFG, German Research Foundation)—Project-ID 73111208- SFB 829 and FOR 5504 project 496,650,118.
- European Joint Project on Rare Diseases, RD20-113, acronym TC-NER.
- The Olav Thon Stiffelsen Prize (2017).

## Version 1 11/17/2025

Electronic publication
